# Feminine hygiene practices among female patients and nurses in Lebanon

**DOI:** 10.1186/s12978-016-0182-4

**Published:** 2016-05-23

**Authors:** Elie Attieh, Samer Maalouf, Dina Roumieh, Pamela Abdayem, Georges AbiTayeh, Assaad Kesrouani

**Affiliations:** Obstetrics and Gynecology Department, Faculty of Medicine, Saint Joseph University, Hotel-Dieu de France Hospital, PO Box: 166830, Adib Ishac Street, Achrafie, Beirut, Lebanon; Faculty of Medicine, Saint Joseph University, Beirut, Lebanon

**Keywords:** Hygiene, Vaginal infections, Vaginal douching, Vaginitis

## Abstract

**Background:**

Inappropriate feminine hygiene practices are related to vulvovaginitis. We investigated the prevalence of personal hygiene habits among Lebanese women as well as their awareness of adequate practices.

**Methods:**

Consists of a cross-sectional observational study. Female patients and nurses at Hotel-Dieu de France University Hospital in Beirut- Lebanon filled a questionnaire about their intimate hygiene habits and knowledge of proper practices.

**Results:**

The study included 249 women. 21.3 % of the 136 nurses and 38.9 % of the 113 patients reported a history of vulvovaginitis. The majority of women took an intimate bath at least twice daily. 14 % of nurses and 17 % of patients douched.20. Seven percent of the nurses and 43.4 % of the patients used wet wipes. 1.5 % of nurses and 4.4 % of patients used feminine deodorant sprays. There was a significant lack of awareness mainly among patients about suitable hygiene practices as well for their adverse effects.

**Conclusion:**

Education provided by nurses, and other healthcare providers is essential to promote reproductive health among Lebanese women.

## Background

Vulvovaginitis, a widespread disorder of the vagina accounts for approximately 10 million office visits to gynecologists every year in the United States (US) [1]. In a random digit dialing survey, 8 percent of Caucasian women and 18% of African-American women in the US reported an episode of vaginal symptoms in the previous year [2]. Symptoms include pruritus, erythema, pain, vulvar excoriations as well as changes in the color or odor or volume of vaginal discharge [3]. Studies indicated that each practice disrupting the normal vaginal ecosystem leads to the development of vulvovaginitis [4].

Perceptions about reproductive health issues differ significantly between countries, societies, and individuals. Socioeconomic status, race, religion and level of education influence women’s perceptions and behaviors regarding their reproductive health. In particular, feminine hygiene practices vary among women with a high prevalence of erroneous behaviors that predispose them to vulvovaginitis.

While few studies about certain feminine hygiene practices, especially vaginal douching, have been conducted in the US and in countries such as Egypt and Turkey, information about intimate hygiene practices among Lebanese women is lacking. Hence, we conducted a cross-sectional study to determine the prevalence of several intimate hygiene habits and their level of knowledge in a population of Lebanese women including nurses and patients. Our aim is to assess the level of public awareness about this subject and the subsequent need for educational interventions to optimize reproductive health in Lebanon.

## Methods

A cross-sectional observational study was conducted at Hotel-Dieu de France University Hospital in Beirut, Lebanon, between January and April 2014. The study population included all female registered nurses at the hospital as well as all the female patients admitted to the department of Obstetrics and Gynecology. There were no exclusion criteria except illiteracy.

Hotel-Dieu de France’s ethical committee approved the study protocol. Investigators provided information about the study to all participants. Oral informed consent is mandatory for all participants before answering the questionnaire that includes a four-page questionnaire, available in French and Arabic languages. The Arabic translation is tested on a population of 10 medical residents and declared identical in meaning to the original French version. Participants were given privacy and time to complete the questionnaire without any disturbance or discomfort.

The questionnaire included three sections. The first section collected socio-demographic data as well as general medical history and reproductive history. The second section inquired about intimate hygiene habits, contraception and vulvovaginitis symptoms, while the third one evaluated women’s awareness about adequate practices by means of yes or no questions. We assessed mainly the frequency of intimate wash, vaginal douching, use of wipes and deodorants, pubic hair removal and vulvovaginitis.

The collected data was analyzed using Statistical Package for Social Science software (SPSS, version 20).

## Results

One hundred and forty-seven patients and 330 nurses were asked to complete the questionnaire between January and April 2014. One hundred and thirteen patients (76.8 %) and 136 nurses (41.2 %) agreed to participate. The mean age of patients was 41.8 and of nurses was 32.4 . The first population selected consists of nurses at Hotel-Dieu de France Hospital, who are predominantly Christian (94.1 %) and reside in urban areas with half of them being married. The second population includes female patients admitted to the Obstetrics and Gynecology department at Hotel-Dieu de France Hospital who are predominantly married (92 %), mostly Christian (69 %), housewives (54.9 %) with a university education (59.3 %). Socio-demographic characteristics of the studied population are listed in Table 1.

**Table 1:** Socio-demographic characteristics of the studied population

Category	Sub-Category	Nurses (n = 136)	Patients (n = 113)	p
Education	Middle School	0 (0 %)	11 (9.7 %)	0.0001
	High School	1 (0.7 %)	28 (24.8 %)	
	University	135 (99.3 %)	67 (59.3 %)	
Profession	Student	1 (0.7 %)	1 (0.9 %)	0.0001
	Employee	131 (96.3 %)	43 (41.1 %)	
	Independent	3 (2.2 %)	6 (5.3 %)	
	House wife	0 (0 %)	62 (54.9 %)	
Civil status	Single	52 (38.2 %)	7 (6.2 %)	0.0001
	In a relationship	7 (5.1 %)	0 (0 %)	
	Married	76 (55.9 %)	104 (92 %)	
	Divorced/Widowed	1 (0.7 %)	1 (1.8 %)	
Religion	Christian	128 (94.1 %)	78 (69 %)	0.001
	Muslim	5 (3.7 %)	21 (18.6 %)	
	Druze	1 (0.7 %)	2 (1.8 %)	
	Other	2 (1.5 %)	0 (0 %)	

Twenty-nine nurses (21.3 %) had a history of vaginitis and 5 (17.2 %) were treated more than once yearly, whereas 44 patients (38.9 %) had a history of vaginitis and 11 (25 %) had recurrent vaginitis. The most commonly reported symptoms of vulvovaginitis were pruritus (8.8 % of the nurses and 9.7 % of the patients) and burning sensation (3.7 % of the nurses and 2.7 % of the patients).

The majority of women took an intimate bath at least twice daily (90.5 % of the nurses versus 84.1 % of the patients. Table 2 details various reasons for intimate bathing among nurses and patients.

**Table 2:** Reasons for intimate bathing among nurses and patients

Reasons for intimate bathing	Nurses	Patients	p-value
To feel fresh	114 (83.8 %)	97 (85.8 %)	0.93
Before sports	24 (17.6 %)	11 (9.8 %)	0.0001
After sports	80 (58.8 %)	26 (23.2 %)	0.0001
Before sexual intercourse	66 (48.5 %)	56 (50 %)	0.00008
After sexual intercourse	78 (57.4 %)	56 (49.6 %)	0.000003
After defecation	66 (48.5 %)	52 (46.4 %)	0.000027
After miction	39 (28.7 %)	45 (39.8 %)	0.00048
Before menstruation	40 (29.4 %)	38 (33.6 %)	0.000003
After menstruation	94 (69.1 %)	71 (63.4 %)	0.000134
To prevent infections	84 (61.8 %)	55 (49.1 %)	0.0001
To treat infections	65 (47.8 %)	19 (16.8 %)	0.0001
To get rid of vaginal discharge	81 (59.6 %)	31 (27.4 %)	0.0001
To get rid of vaginal odor	86 (63.2 %)	36 (31.9 %)	0.0001
Relief from itching	75 (55.1 %)	22 (19.5 %)	0.0001

Vaginal douching was common for 14 % of nurses and 15 % of patients. They used plain water (33 %), commercial solutions (22 %), bicarbonate-based solutions (22 %) and soap and water (20 %).

The two groups used a similar method to insert the liquid products inside the vagina (mostly sponges). Sixty-six percent of patients reported daily basis douching while 60 % of nurses douched only once weekly. Twice daily intimate washing is more frequent among nurses. They undergo intimate washing more frequently before and after sports, after sexual intercourse, after defecation, after menstruation and in the case of infections, discharge, foul odors or pruritus. On the other hand, washing before intercourse, before menstruation and after urination are more common in the patient group.

The use of wet wipes for intimate cleaning was reported in 20.7 % of the nurses and 43.4 % of the patients. No significant difference was found between the two groups regarding the use of vaginal deodorant sprays (1.5 % of nurses versus 4.4 % of patients,). As for other adequate hygiene practices, both patients and nurses seem to be aware of the utility of wearing cotton underwear and avoiding tight-fitting clothes to prevent trapping of sweat, bacterial and fungal multiplication and subsequent vaginitis. Moreover, 60.2 % of the patients and 35.3 % of nurses do not know that tampons should be changed at least twice a day regardless of the menstrual flow to decrease vaginal discharge and to prevent odors, as well as bacterial and fungal growth. The women in our study recognize signs of vaginal infections and are aware that white or yellow vaginal discharge can be normal.

Regular pubic hair removal was similar between the two populations (78.7 % of nurses versus 71.7 % of patients,). 13.2 % of the nurses and 17.7 % of the patients reported subsequent irritation with the majority of the reactions resulting from the use of wax (5.9 % of nurses and 10.6 % of patients) and shaving cream (9.6 % of nurses and 3.5 % of patients).

The extent of knowledge of the two populations regarding reproductive health issues, particularly feminine hygiene practices is different regarding vaginal douche, deodorant spray and how to wipe after defecation and is fully detailed in Table 3.

**Table 3:** Extent of knowledge regarding reproductive health issues in females patients and nurses

		Yes	No	Do not know	p-value
Spray deodorant gets rid of vaginal odor if used regularly	Nurses	6.7 %	49.6 %	42.2 %	0.696
	Patients	4.4 %	46.0 %	46.9 %	
	Total	5.6 %	48.0 %	44.4 %	
Spray deodorant can replace the use of soap and water	Nurses	0.7 %	80.9 %	17.6 %	0.001
	Patients	0.9 %	56.6 %	39.8 %	
	Total	0.8 %	69.9 %	27.7 %	
Spray deodorant should be applied as intra-vaginally as possible	Nurses	0.7 %	64.7 %	31.6 %	0.141
	Patients	0.0 %	52.2 %	45.1 %	
	Total	0.4 %	59.0 %	37.8 %	
Vaginal douching is recommended whenever there is a change in vaginal discharge	Nurses	32.4 %	27.9 %	36.8 %	0.003
	Patients	12.4 %	36.3 %	47.8 %	
	Total	23.3 %	31.7 %	41.8 %	
Vaginal douching is recommended after each sexual intercourse	Nurses	36.8 %	40.4 %	22.1 %	0.0001
	Patients	15.0 %	41.6 %	40.7 %	
	Total	26.9 %	41.0 %	30.5 %	
Vaginal douching is recommended after menstruation	Nurses	27.9 %	47.1 %	21.3 %	0.001
	Patients	13.4 %	39.3 %	43.8 %	
	Total	21.4 %	43.5 %	31.5 %	
Vaginal douching can hurt the vaginal mucosa	Nurses	52.2 %	12.5 %	33.1 %	0.004
	Patients	29.2 %	18.6 %	49.6 %	
	Total	41.8 %	15.3 %	40.6 %	
Vaginal douching should not be done unless recommended by a doctor	Nurses	63.2 %	18.4 %	16.2 %	0.0001
	Patients	30.1 %	18.6 %	48.7 %	
	Total	48.2 %	18.5 %	30.9 %	
Vaginal douching causes a cystocele	Nurses	2.9 %	36.0 %	59.6 %	0.036
	Patients	1.8 %	20.4 %	73.5 %	
	Total	2.4 %	28.9 %	65.9 %	
Regular application of antiseptics prevents vaginal infections	Nurses	21.3 %	56.6 %	19.1 %	0.223
	Patients	16.8 %	50.4 %	30.1 %	
	Total	19.3 %	53.8 %	24.1 %	
Cotton underwear prevent vaginal infections	Nurses	65.2 %	24.4 %	9.6 %	0.143
	Patients	70.8 %	15.0 %	10.6 %	
	Total	67.7 %	20.2 %	10.1 %	
Regular vaginal douching prevents vaginal infections	Nurses	34.6 %	41.2 %	23.5 %	0.007
	Patients	18.6 %	39.8 %	38.9 %	
	Total	27.3 %	40.6 %	30.5 %	
Tight-fitting clothes prevent vaginal infections	Nurses	6.6 %	77.9 %	14.0 %	0.136
	Patients	10.6 %	65.5 %	19.5 %	
	Total	8.4 %	72.3 %	16.5 %	
Wiping after miction/defecation must be done from front to rear	Nurses	93.4 %	1.5 %	4.4 %	0.0003
	Patients	77.0 %	4.4 %	14.2 %	
	Total	85.9 %	2.8 %	8.8 %	
Vaginal sprays eradicate bacteria responsible for vaginitis	Nurses	3.7 %	56.6 %	39.0 %	0.0001
	Patients	15.0 %	29.2 %	53.1 %	
	Total	8.8 %	44.2 %	45.5 %	
Sprays are strictly harmless products	Nurses	2.9 %	55.9 %	40.4 %	0.646
	Patients	3.5 %	52.2 %	41.6 %	
	Total	3.2 %	54.2 %	41.0 %	
Allergic or irritative reactions to deodorants may occur evenif their previous use did not cause any problem	Nurses	56.6 %	14.0 %	26.5 %	0.0001
	Patients	28.3 %	18.6 %	50.4 %	
	Total	43.8 %	16.1 %	37.3 %	
Allergic or irritative reactions to deodorants do not require medical follow-up	Nurses	5.1 %	79.4 %	14.7 %	0.0001
	Patients	6.2 %	45.1 %	46.0 %	
	Total	5.6 %	63.9 %	28.9 %	
Modification of the quantity of vaginal discharge without signs of infection does not occur in healthy women	Nurses	30.9 %	55.1 %	13.2 %	0.002
	Patients	22.1 %	43.4 %	31.9 %	
	Total	26.9 %	49.8 %	21.7 %	
Persistent white to yellow vaginal discharge in small quantities is normal	Nurses	69.1 %	17.6 %	11.8 %	0.001
	Patients	46.0 %	22.1 %	28.3 %	
	Total	58.6 %	19.7 %	19.3 %	
Tampons should be changed twice daily regardless of the blood flow, to prevent blocking of normal vaginal discharge	Nurses	40.4 %	22.8 %	35.3 %	0.0001
	Patients	20.4 %	15.9 %	60.2 %	
	Total	31.3 %	19.7 %	46.6 %	
A genital infection in the partner is not considered a source of vaginal infection	Nurses	8.8 %	80.9 %	9.6 %	0.119
	Patients	10.6 %	69.0 %	17.7 %	
	Total	9.6 %	75.5 %	13.3 %	

## Discussion

Vulvovaginitis is a common gynecologic condition. Many preventable causes of vaginitis are related to feminine hygiene practices, which vary considerably by race, age, religion and cultural origins.

The main findings of this study include describing several common feminine hygiene habits among Lebanese women and assessing the level of awareness about adequate hygiene practices.

The prevalence of vaginitis and recurrent vaginitis in this study is significantly lower among nurses, which emphasizes the role of education in the prevention of this condition.

Vaginal douching by a liquid solution in the vagina in the aim of cleanliness, odor control, or relief of vaginal itching and irritation is a common practice worldwide especially among African American and Muslim women [4]. The use of douching is similar between our two groups (14 % of nurses and 15.1 % of patients) and is considerably less than the prevalence reported by Shaaban et al. in Egypt (73 %), Erbil et al. (38.6 %) and Ege et al. (61.5 %) in Turkey [5–7]. This difference could be explained by the fact that the majority of the women in our study are Christians and do not douche for religious reasons as in other countries in the Middle-East. Thirty-seven percent of women reported douching in a study by Aral et al. in a nationally representative sample of 8450 women between the ages of 15 and 44 years reported that 37 % of them used douching [8]. Another cross-sectional study by Cottrel et al. showed that 76 % of 483 women in Florida douched, 43 % douched once per month, and 36 % were unaware they should not douche [9]. Our findings are different from US studies. This could be explained by the high prevalence of douching among African American women (27.7 % of African American women versus 9.1 % of white women and 15 % of Hispanic women) and among women who have multiple sexual partners [10, 11]. Low socioeconomic status and lack of education are also associated with increased douching [12]. The high rate of college education among women may also explain the low percentage of douching. Half the nurses (52.2 %) know that douching may injure the vaginal mucosa and 63.2 % state that douching should be performed per doctor’s order, yet a third of them still mention indications for douching. On the other hand, a greater number of female patients responded “Do not know” to the questions about the indications for douching. Douching should be discouraged because it alters the vaginal flora and predisposes women to bacterial vaginosis, pelvic inflammatory disease, endometritis and sexually transmitted infections [13, 14]. Ott et al. examined the association between the use of feminine hygiene products (wipes, sprays, douches and yeast creams) and sexually transmitted infections (STI). Twenty-seven percent of the 295 recruited adolescent women used feminine wipes and this was related to a recent STD [15]. Feminine wipe use may irritate the vulvar skin and predispose to vulvar dermatitis. Our results show a widespread use of wipes among patients which could be due to lack of information. A small percentage of patients (4.4 %) and nurses (1.5 %) reported using intimate deodorant sprays, which is less than the 29 % reported by Ott et al.[15] probably due to the lack of publicity about these products in our country. In addition, there is an obvious lack of information mainly among patients regarding the potential harm resulting from their use (vulvar dermatitis, allergic reactions…) [16].

Women often erroneously perceive their personal practices as safe since they become used to it. Marin et al. reported that over 60 % of 530 women attending a specialty clinic for vulvar diseases, reported adverse intimate hygiene practices which is somehow similar to the results we obtained [17]. Education about adequate feminine hygiene practices is therefore crucial to improve Lebanese women’s reproductive health, Nurses play a crucial role in promoting reproductive health by correcting false information about feminine hygiene.

One of the limitations of our study is the selection bias, the majority of women being married, Christian and of higher education level. However, one would argue that the lack of awareness about adequate practices demonstrated among educated women would also be present among less-educated women. Being an observational study represents a limitation to attain solid conclusions. It would be useful to perform a similar cross-sectional study on a sample of women admitted to hospitals all over Lebanon, and to compare the results obtained before and after educational campaigns, personal hygiene classes or other informative interventions. Our study represents a pilot study in Lebanon and could lead to metaanalyses or larger comparative studies that would enrich our knowledge of feminine hygiene.

## Conclusion

Lack of information about adequate feminine hygiene practices is common among Lebanese women. Nurses seem to have more appropriate practice and knowledge about this issue than patients. Education provided by health care providers is important to optimize reproductive health in Lebanon.
